# Proteomic Study of the Interactions between Phages and the Bacterial Host *Klebsiella pneumoniae*

**DOI:** 10.1128/spectrum.03974-22

**Published:** 2023-03-06

**Authors:** Inés Bleriot, Lucia Blasco, Olga Pacios, Laura Fernández-García, María López, Concha Ortiz-Cartagena, Antonio Barrio-Pujante, Felipe Fernández-Cuenca, Álvaro Pascual, Luis Martínez-Martínez, Jesús Oteo-Iglesias, María Tomás

**Affiliations:** a Microbiology Translational and Multidisciplinary (MicroTM)-Research Institute Biomedical A Coruña (INIBIC) and Microbiology Department of Hospital A Coruña (CHUAC), University of A Coruña (UDC), A Coruña, Spain; b Clinical Unit of Infectious Diseases and Microbiology, Hospital Universitario Virgen Macarena, Institute of Biomedicine of Seville (University Hospital Virgen Macarena/CSIC/University of Seville), Seville, Spain; c Clinical Unit of Microbiology, Reina Sofía University Hospital, Department of Agricultural Chemistry, Edaphology and Microbiology, University of Cordoba, Maimonides Biomedical Research Institute (IMIBIC), Cordoba, Spain; d Reference and Research Laboratory for Antibiotic Resistance and Health Care Infections, National Centre for Microbiology, Institute of Health Carlos III, Majadahonda, Madrid, Spain; e CIBER de Enfermedades Infecciosas (CIBERINFEC), Instituto de Salud Carlos III, Madrid, Spain; Barnard College, Columbia University

**Keywords:** *Klebsiella pneumoniae*, lytic phage, phage-host interaction, defense mechanism, prophage, plasmid, *Klebsiella*, bacteriophage evolution, bacteriophage, virus-host interactions

## Abstract

Phages and bacteria have acquired resistance mechanisms for protection. In this context, the aims of the present study were to analyze the proteins isolated from 21 novel lytic phages of Klebsiella pneumoniae in search of defense mechanisms against bacteria and also to determine the infective capacity of the phages. A proteomic study was also conducted to investigate the defense mechanisms of two clinical isolates of K. pneumoniae infected by phages. For this purpose, the 21 lytic phages were sequenced and *de novo* assembled. The host range was determined in a collection of 47 clinical isolates of K. pneumoniae, revealing the variable infective capacity of the phages. Genome sequencing showed that all of the phages were lytic phages belonging to the order *Caudovirale*s. Phage sequence analysis revealed that the proteins were organized in functional modules within the genome. Although most of the proteins have unknown functions, multiple proteins were associated with defense mechanisms against bacteria, including the restriction-modification system, the toxin-antitoxin system, evasion of DNA degradation, blocking of host restriction and modification, the orphan CRISPR-Cas system, and the anti-CRISPR system. Proteomic study of the phage-host interactions (i.e., between isolates K3574 and K3320, which have intact CRISPR-Cas systems, and phages vB_KpnS-VAC35 and vB_KpnM-VAC36, respectively) revealed the presence of several defense mechanisms against phage infection (prophage, defense/virulence/resistance, oxidative stress and plasmid proteins) in the bacteria, and of the Acr candidate (anti-CRISPR protein) in the phages.

**IMPORTANCE** Researchers, including microbiologists and infectious disease specialists, require more knowledge about the interactions between phages and their bacterial hosts and about their defense mechanisms. In this study, we analyzed the molecular mechanisms of viral and bacterial defense in phages infecting clinical isolates of K. pneumoniae. Viral defense mechanisms included restriction-modification system evasion, the toxin-antitoxin (TA) system, DNA degradation evasion, blocking of host restriction and modification, and resistance to the abortive infection system, anti-CRISPR and CRISPR-Cas systems. Regarding bacterial defense mechanisms, proteomic analysis revealed expression of proteins involved in the prophage (FtsH protease modulator), plasmid (cupin phosphomannose isomerase protein), defense/virulence/resistance (porins, efflux pumps, lipopolysaccharide, pilus elements, quorum network proteins, TA systems, and methyltransferases), oxidative stress mechanisms, and Acr candidates (anti-CRISPR protein). The findings reveal some important molecular mechanisms involved in the phage-host bacterial interactions; however, further study in this field is required to improve the efficacy of phage therapy.

## INTRODUCTION

Bacteriophages, or phages, are natural predators of bacteria. Phages are the most abundant and ubiquitous biological entities on Earth, accounting for an estimated total of 10^31^ viral particles (1, 2). In the current context of increasing antibiotic resistance, the emergence of alternative therapies is welcome. Thus, lytic phages are currently considered one of the best options for treating infections caused by multidrug-resistant (MDR) bacteria (3, 4), as demonstrated in clinical trials conducted to date (5–10). In general, phage therapy has some advantages over the use of conventional antibiotics, such as low toxicity and high host specificity (3, 11). Another characteristic of phages that makes them good candidates for therapy is their ability to adapt to changes in the bacterial host, which has resulted from the coevolution of both types of organisms (12).

Bacteria have developed mechanisms to prevent phage infection at almost all stages of the viral replication cycle (13, 14). First, bacteria can prevent phage attachment by mutating or altering their surface receptors, by producing inhibitors that outcompete the phage for receptors, or by producing polysaccharides that physically mask phage receptors (13). In order to prevent injection of phage DNA into the cytoplasm, the bacteria then use the superinfection exclusion system, which is characterized by proteins that block the entry of phage DNA into host cells (15). If the phage nevertheless manages to enter the cell, the bacteria employ other defense systems such as the abortive infection system (Abi) to interrupt phage development at any stage (replication, transcription, or translation) (16), the bacteriophage exclusion system (17) or the defense island system associated with restriction-modification (18) to interrupt replication. Bacteria can also use their toxin-antitoxin (TA) systems, which often lead to reduced bacterial metabolism and phage inhibition (19–21), when the antitoxin is degraded under stress conditions by the protease system (22). Bacteria can also employ other types of systems to cleave phage DNA, such as the restriction-modification (RM) system and a cognate DNA methylase, which modifies and protects the host DNA (23). They can also employ the clustered regularly interspaced short palindromic repeats–CRISPR-associated proteins (CRISPR-Cas) system, an adaptive immune system (24) characterized by the acquisition of spacer sequences, which are small fragments of foreign nucleic acids of phage or foreign DNA, between the repeats of the CRISPR locus (25).

Phages have, in turn, developed counterstrategies to evade bacterial defense mechanisms (26). For example, for successful adsorption, phages can modify their receptor-binding proteins by acquiring mutations to obtain new receptors (27). In turn, they are also able to acquire enzymes such as depolymerase to access masked receptors (28, 29) in a way that allows them to interact with a surface component expressed by the host at that time (30). However, when a phage genome manages to enter the cell, it can still face the myriad intracellular antiviral barriers described above. Phages can respond to these by promoting the mutation of specific genes to prevent activation of the bacterial Abi system (31). They can also evade bacterial RM systems by reducing the number of restriction sites in their genome (32), modifying bases in their genome (33), coinjecting protein (for instance, DarA and DarB in the phage P1) with the genome to bind directly to the phage DNA and mask restriction sites (34), stimulating the action of modification enzymes and degrading an RM cofactor. Phages can also sequester host antitoxins via a protein that probably inhibits Lon protease activity to avoid the deleterious action of the toxins of the TA systems (35). To circumvent the effect of bacterial TA systems, one phage, T4, encodes its own antitoxin protein (Dmd) that functionally replaces the unstable antitoxin of the host, thereby promoting phage propagation (36). Finally, phages have developed mechanisms to evade the bacterial CRISPR-Cas system. For instance, through a single-nucleotide substitution or a complete deletion in the protospacer region or in the conserved protospacer-adjacent motif (37). Phages have also developed anti-CRISPR systems, which basically consist of Acr proteins (typically small proteins of 80 to 150 amino acids) that inhibit bacterial CRISPR-Cas activity by binding directly to, and thus inactivating, the Cas protein, so that phages can successfully replicate in the bacterial host (38).

A better understanding of phage-host interaction could lead to the development of more successful therapeutic applications for phages. In this context, the aims of the present study were to analyze the proteins isolated from 21 novel lytic phages of K. pneumoniae in search of defense mechanisms against bacteria and also to determine their infective capacity. In addition, the other aim of this work was to investigate the defense mechanisms of bacteria in response to phage infection.

## RESULTS

### Isolation, propagation, and electron microscopic analysis of phages.

**FIG 1:**
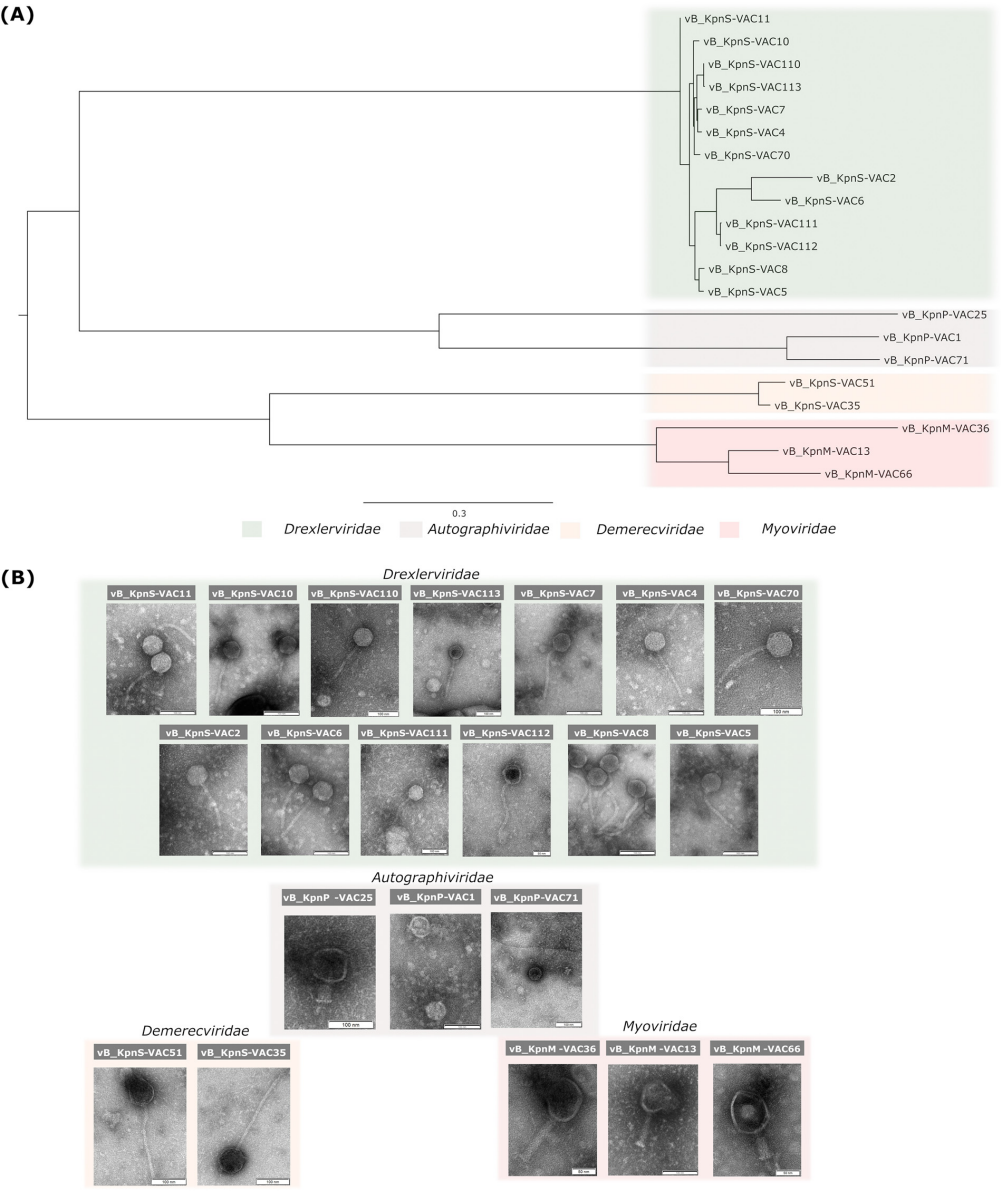
(A) Phylogenetic analysis of 21 phages performed with the nucleotide sequence of the large terminase subunit of each phage. (B) TEM images showing the structure of the 21 phages under study. All belong to the order *Caudovirales.* vB_KpnS-VAC2, vB_KpnS-VAC4, vB_KpnS-VAC5, vB_KpnS-VAC6, vB_KpnS-VAC7, vB_KpnS-VAC8, vB_KpnS-VAC10, vB_KpnS-VAC11, vB_KpnS-VAC35, vB_KpnS-VAC70, vB_KpnS-VAC110, vB_KpnS-VAC111, vB_KpnS-VAC112, and vB_KpnS-VAC113 are characterized by large and flexible tails. On the other hand, phages vB_KpnP-VAC1, vB_KpnP-VAC25, and vB_KpnP-VAC71 are characterized by a short tail. Finally, phages vB_KpnM-VAC13, vB_KpnM-VAC36, and vB_KpnM-VAC66 are characterized by an icosahedral capsid and a rigid, contractile tail. The TEM scale bar represents 50 or 100 nm, depending on the phage.

The 21 phages examined in this study, named according to accepted practices (39) (Table 1), were obtained from wastewater samples. However, to facilitate reading this article, we will incorporate the data previously found in these studies in order to enable comparison between the phages. Thus, transmission electron microscopy (TEM) studies revealed, according to the morphology, that 15 phages have long and flexible tails (vB_KpnS-VAC2, vB_KpnS-VAC4, vB_KpnS-VAC5, vB_KpnS-VAC6, vB_KpnS-VAC7, vB_KpnS-VAC8, vB_KpnS-VAC10, and vB_KpnS-VAC11 [20]; and vB_KpnS-VAC35, vB_KpnS-VAC51, vB_KpnS-VAC70, vB_KpnS-VAC110, vB_KpnS-VAC111, vB_KpnS-VAC112, and vB_KpnS-VAC113). Moreover, three (vB_KpnM-VAC13 [40], vB_KpnM-VAC36, and vB_KpnM-VAC66 [41]) present icosahedral capsid and a rigid, contractile tails. Finally, three phages have small and noncontractile tails (vB_KpnP-VAC1 [20], vB_KpnP-VAC25, and vB_KpnP-VAC71) (Fig. 1B).

**TABLE 1:** Characteristics of 21 K. pneumoniae lytic phages^*a*^

Phage	Natural host	GenBank accession no.		Family	Genus	Genome size (bp)	%G+C	Source or reference
		Biosample	Nucleotide					
vB_KpnP-VAC1	ATCC 10031	SAMN19773206	MZ428229.1	*Autographiviridae*	*Teetrevirus*	39,371	50.75	20
vB_KpnS-VAC2	ATCC 10031	SAMN19773207	MZ428221.1	*Drexlerviridae*	*Webervirus*	51,784	47.86	20
vB_KpnS-VAC4	ATCC 10031	SAMN19773215	MZ428222.1	*Drexlerviridae*	*Webervirus*	45,558	51.11	20
vB_KpnS-VAC5	ATCC 10031	SAMN19773216	MZ428223.1	*Drexlerviridae*	*Webervirus*	49,636	50.46	20
vB_KpnS-VAC6	ATCC 10031	SAMN19773219	MZ428224.1	*Drexlerviridae*	*Webervirus*	51,554	51.63	20
vB_KpnS-VAC7	ATCC 10031	SAMN19773224	MZ428225.1	*Drexlerviridae*	*Webervirus*	49,684	51.25	20
vB_KpnS-VAC8	ATCC 10031	SAMN19773221	MZ428226.1	*Drexlerviridae*	*Webervirus*	48,933	50.52	20
vB_KpnS-VAC10	ATCC 10031	SAMN19773232	MZ428227.1	*Drexlerviridae*	*Webervirus*	48,935	50.65	20
vB_KpnS-VAC11	ATCC 10031	SAMN19773540	MZ428228.1	*Drexlerviridae*	*Webervirus*	48,826	50.83	20
vB_KpnM-VAC13	ATCC 10031	SAMN22059222	MZ322895.1	*Myoviridae*	*Slopekvirus*	174,826	41.93	40
vB_KpnP-VAC25	K3579	SAMN20298869	MZ571827.1	*Autographiviridae*	*Drulisivirus*	43,777	53.76	This study
**vB_KpnS-VAC35**	**K3574**	SAMN20298871	MZ571828.1	***Demerecviridae***	***Sugarlandvirus***	**112,862**	**45.44**	**This study**
**vB_KpnM-VAC36**	**K3573**	SAMN20298872	MZ571829.1	***Myoviridae***	***Marfavirus***	**169,970**	**40.90**	**This study**
vB_KpnS-VAC51	K3325	SAMN23489160	MZ571830.1	*Demerecviridae*	*Sugarlandvirus*	113,149	45.40	This study
vB_KpnM-VAC66	K3320	SAMN22059211	MZ612130.1	*Myoviridae*	*Slopekvirus*	178,532	41.72	41
vB_KpnS-VAC70	K3318	SAMN20298916	MZ571831.1	*Drexlerviridae*	*Webervirus*	49,631	50.70	This study
vB_KpnP-VAC71	K3318	SAMN20298917	MZ571832.1	*Autographiviridae*	*Przondovirus*	40,388	52.98	This study
vB_KpnS-VAC110	K2691	SAMN24377650	OM032871.1	*Drexlerviridae*	*Webervirus*	45,195	50.44	This study
vB_KpnS-VAC111	K2691	SAMN20298918	ON881905.1	*Drexlerviridae*	*Webervirus*	50,559	50.50	This study
vB_KpnS-VAC112	K2691	SAMN20298919	MZ571833.1	*Drexlerviridae*	*Webervirus*	49,068	50.20	This study
vB_KpnS-VAC113	K2691	SAMN20298921	MZ571834.1	*Drexlerviridae*	*Webervirus*	49,568	50.46	This study

a Phages selected for phage interaction studies are indicated in boldface.

### Phage genome annotation.

**(i) Phage genome analysis.** The phage genome sequencing revealed that all phages under study, available from the GenBank BioProject PRJNA739095 (Table 1), were lytic *Caudovirales* phages, i.e., dsDNA tailed phages, lacking lysogenic genes such as integrase, recombinase, and excisionase. More specifically, 61.90% of the phages belonged to the *Drexerviridae* family (13 of 21 phages), 14.29% belonged to the *Autographiviridae* family (3 of 21 phages), 14.29% belonged to the *Myoviridae* family (3 of 21 phages), and 9.52% belonged to the *Demerecviridae* family (2 of 21 phages). The genomes ranged from 39,371 bp in the case of phage vB_KpnP-VAC1, a member of the family *Autographiviridae* and the genus *Teetrevirus*, to 178,532 bp in the case of phage vB_KpnM-VAC66, a member of the family *Myoviridae*, and the genus *Slopekvirus*. The guanine-cytosine content ranged from 40.90% in the case of vB_KpnM-VAC36 to 53.76% in the case of vB_KpnP-VAC25. The genomic study revealed that the structure of the phage genomes varied depending on the type of phage. In the case of the members of the *Drexerviridae* (vB_KpnS-VAC2-11, vB_KpnS-VAC70, and vB_KpnS-VAC110-113), *Autographiviridae* (vB_KpnP-VAC1, vB_KpnP-VAC25, and vB_KpnP-VAC71), and *Demerecviridae* (vB_KpnS-VAC35 and vB_KpnS-VAC51) families, the genome was organized in functional modules of genes related to structure, packaging, lysis, transcription, and regulation. In contrast, for members of the *Myoviridae* (vB_KpnM-VAC13, vB_KpnM-VAC36, and vB_KpnM-VAC66) family, which are “larger phages” (>100 bp), no lysis-specific blocks were distinguished, and structural and morphogenesis-related proteins were repeated in several blocks throughout the genome. Considering the lysis genes, all phages had endolysins and holins, proteins which are responsible for the degradation of the bacterial cell wall during infection of the host. However, a difference was observed in terms of spanin, a protein involved in the lysis process in Gram-negative hosts, depending on the family to which the phage belongs: the *Drexerviridae* family had a unimolecular spanin (U-spanin), while the *Autographiviridae*, *Demerecviridae*, and *Myoviridae* families had a heterodimer molecule of spanin (I-spanin and O-spanin). Regarding the depolymerase genes, generally related to the tail receptor, five of the phages (23.81%) had one depolymerase (vB_KpnS-VAC4, vB_KpnS-VAC7, vB_KpnS-VAC10, vB_KpnS-VAC11, and vB_KpnS-VAC70), while two of the phages (9.52%) (vB_KpnS-VAC2 and vB_KpnS-VAC6) had two different depolymerase genes. It has been observed that the “larger phages” (vB_KpnM-VAC13, vB_KpnS-VAC35, vB_KpnM-VAC36, vB_KpnS-VAC51, and vB_KpnM-VAC66) had numerous tRNA genes (1, 21, 7, 22, and 1, respectively). The presence of HNH homing endonucleases has been observed in the genomes of three phages (vB_KpnP-VAC1, vB_KpnM-VAC13, and vB_KpnM-VAC66). The phage vB_KpnM-VAC66 contains the highest number of HNH homing endonucleases.

**(ii) Genetic defense mechanisms of phages.** An in-depth study of the phage genomes with different bioinformatic tools revealed the presence of defense mechanisms (Table 2): (i) 35 RM system evasion mechanisms located in 16 phages, (ii) six TA systems located in two phages (vB_KpnM-VAC13 and vB_KpnM-VAC66), (iii) one DNA degradation evasion located in phage vB_KpnP-VAC1, (iv) four blocking RM of host bacteria located in phage vB_KpnM-VAC36, (v) seven genes that confer resistance to the Abi system of host bacteria located in three phages (vB_KpnM-VAC13, vB_KpnM-VAC36, and vB_KpnM-VAC66), and, finally, (vi) two possible orphan CRISPR-Cas system were located in the phages vB_KpnS-VAC35 and vB_KpnS-VAC51. In addition, almost all phages possessed a possible anti-CRISPR system, composed by Acr and Aca protein, except for phages vB_KpnS-VAC112 and vB_KpnS-VAC113 (see Table S1 in the supplemental material). An inhibitor of the TA system (protein ID QZE51102.1) was also found in the genome of the phage vB_KpnP-VAC1.

**TABLE 2:** Phage defense mechanisms against bacteria detected in the phages under study^*a*^

Phage	E value	Description	Accession no.
Evasion of the RM system			
vB_kpnS-VAC2	1.00E–177	DNA adenine methyltransferase	QZE50413.1
	4.00E–166	DNA methylase	QZE50429.1
	2.00E–47	DNA cytosine methylase	QZE50430.1
	4.00E–47	DNA cytosine methyltransferase	QZE50431.1
vB_KpnS-VAC4	1.00E–171	Cytosine DNA methylase	QZE50543.1
	3.00E–180	DNA adenine methyltransferase	QZE50559.1
vB_KpnS-VAC5	3.00E–179	DNA adenine methyltransferase	QZE50572.1
	4.00E–170	DNA cytosine methyltransferase	QZE50589.1
vB_KpnS-VAC6	2.00E–180	DNA adenine methyltransferase	QZE50665.1
	9.00E–178	DNA methylase	QZE50681.1
vB_KpnS_VAC7	8.00E–171	DNA cytosine methyltransferase	QZE50785.1
	3.00E–180	DNA adenine methyltransferase	QZE50801.1
vB_KpnS-VAC8	2.00E–170	DNA cytosine methyltransferase	QZE50869.1
	3.00E–179	DNA adenine methyltransferase	QZE50885.1
vB_knpS-VAC10	2.00E–41	Methyltransferase type 11	QZE50901.1
	3.00E–179	DNA adenine methyltransferase	QZE50946.1
vB_KpnS-VAC11	1.00E–22	DNA adenine methyltransferase	QZE50991.1
	8.00E–123	DNA adenine methyltransferase	QZE50992.1
	2.00E–173	DNA cytosine methylase	QZE51007.1
vB_KpnM_VAC13	0.00E+00	Putative methyl transferase	QWY13741.1
	0.00E+00	Cytosine-specific methyltransferase	QWY13835.1
vB_KpnM-VAC66	0.00E+00	DNA adenine methylase	QYC51093.1
	0.00E+00	Putative methyl transferase	QYC51156.1
vB_KpnS-VAC70	3.00E–180	DNA adenine methyltransferase	UEW68160.1
	4.00E–169	DNA cytosine methyltransferase	UEW68177.1
vB_KpnS-VAC110	2.10E–164	Cytosine DNA methylase	UKL59162.1
	3.90E–162	DNA adenine methyltransferase	UKL59178.1
vB_KpnS-VAC111	7.00E–179	DNA adenine methyltransferase	UEP19890.1
	3.00E–164	Cytosine DNA methylase	UEP19925.1
vB_KpnS-VAC112	2.00E–41	Methyltransferase type 11	UEW68274.1
	4.00E–169	DNA cytosine methyltransferase	UEW68302.1
	2.00E–180	DNA adenine methyltransferase	UEW68318.1
vB_KpnS-VAC113	5.00E–171	Cytosine DNA methylase	UEP19765.1
	3.00E–179	DNA *N*-6-adenine-methyltransferase	UEP19781.1
			
TA system			
vB_KpnM_VAC13	0.00E+00	RNA ligase/RnlB antitoxin	QWY13674.1
	3.10E–05	DUF1778 domain-containing protein; TA toxin, antitoxin, *N*-acetyltransferase	QWY13811.1
	1.50E–13	RNase III inhibitor; macrodomain, toxin-antitoxin	QWY13854.1
vB_KpnM-VAC66	0.00E+00	RNA ligase/RnlB antitoxin	QYC51086.1
	3.10E–05	DUF1778 domain-containing protein; TA toxin, antitoxin, *N*-acetyltransferase	QYC51228.1
	1.50E–13	RNase III inhibitor; macrodomain, toxin-antitoxin	QYC51272.1
			
Evasion of DNA degradation			
vB_KpnP-VAC1	2.00E–30	Inhibitor of *recBCD* nuclease	QZE51097.1
			
Block RM of host bacteria			
**vB_KpnM-VAC36**	4.00E–159	Anti-restriction nuclease	UEP19370.1
	4.00E–41	Putative anti-restriction nuclease	UEP19372.1
	1.00E–87	Anti-restriction nuclease	UEP19373.1
	0.00E+00	Anti-restriction endonuclease	UEP19375.1
Resistance to Abi system of host bacteria			
vB_KpnM-VAC13	0.00E+00	RIIB protector from prophage-induced early lysis	QWY13711.1
	0.00E+00	RIIA-RIIB membrane associated protein/rIIA lysis inhibitor	QWY13712.1
**vB_KpnM-VAC36**	3.00E+00	RIIA lysis inhibitor	UEP19351.1
	0.00E+00	RIIA membrane-associated protein	UEP19352.1
	0.00E+00	RIIB lysis inhibitor	UEP19353.1
vB_KpnM-VAC66	0.00E+00	RIIB protein	QYC51123.1
	0.00E+00	RIIA protector from prophage-induced early lysis	QYC51124.1
CRISPR-Cas system			
**vB_KpnS-VAC35**		Orphan CRISPR array, 94; no. of spacers, 1	ND
vB_KpnS-VAC51		CRISPR-CAS length, 219; DR length, 25; no. of spacers, 3	ND

a The clinical strains selected for phage interaction studies are indicated in boldface.

**(iii) Phylogenetic analysis of phages.** Phylogenetic analysis was performed by aligning the nucleotide sequence of the large subunit terminase of each phage with MAFF server, followed by the elaboration of the phylogenetic tree with RAxMLHPC-PTHREADS-AVX2 version 8.2.12 (42) under the GTRGAMMA model and 100 bootstrap replicates. This analysis revealed that phages were clustered in the following families: (i) *Drexlerviridae* (vB_KpnS-VAC2, vB_KpnS-VAC4, vB_KpnS-VAC5, vB_KpnS-VAC6, vB_KpnS-VAC7, vB_KpnS-VAC8, vB_KpnS-VAC10, vB_KpnS-VAC11, vB_KpnS-VAC70, vB_KpnS-VAC110, vB_KpnS-VAC111, vB_KpnS-VAC112, and vB_KpnS-VAC113), (ii) *Autographiviridae* (vB_KpnP-VAC1, vB_KpnP-VAC25, and vB_KpnP-VAC71), (iii) *Demerecviridae* (vB_KpnS-VAC35 and vB_KpnS-VAC51), and (iv) *Myoviridae* (vB_KpnM-VAC36, vB_KpnM-VAC13, and vB_KpnM-VAC66). It is worth mentioning that in the case of *Autographiviridae*, phage vB_KpnP-VAC1 was phylogenetically more similar to vB_KpnP-VAC71 than to vB_KpnP-VAC25. Moreover, in the case of the *Myoviridae*, phage vB_KpnM-VAC36, belonging to the genus *Marfavirus*, was phylogenetically more distant than phages vB_KpnM-VAC13 and vB_KpnM-VAC66, which are very similar to each other, as demonstrated in a previous study conducted by our research group, both belonging to the *Slopekvirus* genus (41) (Fig. 1A).

**FIG 2:**
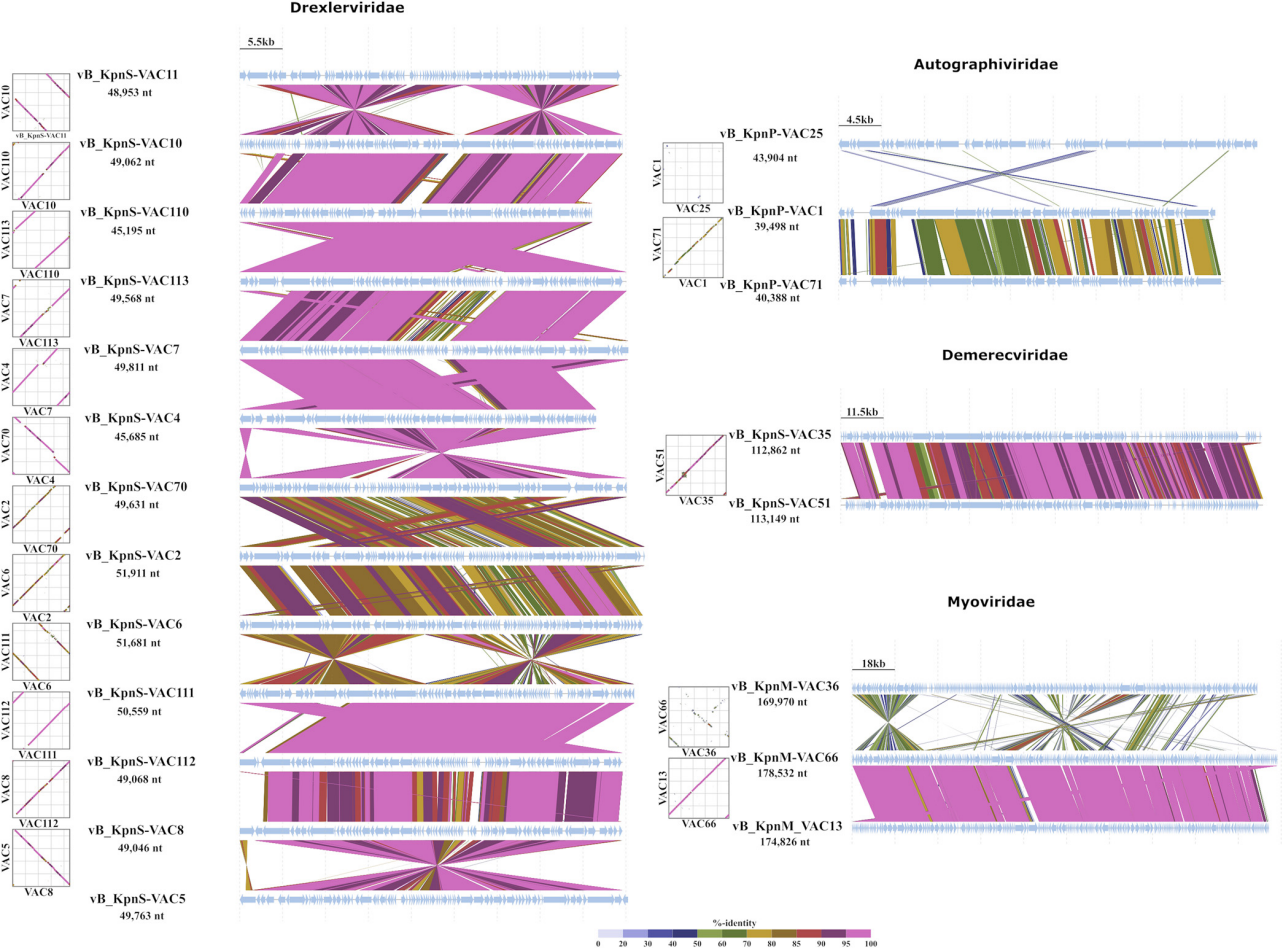
Graphic comparison of the homology of the 21 phages, grouped according to their families and in the same order as in the phylogenetic tree. The schematic representation was conducted with VipTree (https://www.genome.jp/viptree/, accessed in June 2022).

**(iv) Genomic comparison.** In the case of the *Drexlerviridae* family, the results show that phages vB_KpnS-VAC110 and vB_KpnS-VAC113 are very similar (query, 97%; identity, 99.83%), as are phages vB_KpnS-VAC7 and vB_KpnS-VAC4 (query, 91%; identity, 98.93%) and phages vB_KpnS-VAC111 and vB_KpnS-VAC112 (query, 90%; identity, 99.55%). However, phages vB_KpnS-VAC70, vB_KpnS-VAC2, vB_KpnS-VAC6, and vB_KpnS-VAC111 show a low degree of similarity (query, >75%; identity, >82%). In the *Autographiviridae* family, a low degree of similarity between all phages was observed (query, >22%; identity, >76%). In the *Demerecviridae* family, partial similarity between phages vB_KpnS-VAC35 and vB_KpnS-VAC51 (query, 93%; identity, 95.37%) was observed. Finally, in the *Myoviridae* family, the phage vB_KpnM-VAC36 member of the *Marfavirus* genus was very different from the other two phages, member of the *Slopekvirus* family, vB_KpnM-VAC13 (query, 0%; identity, 80.75%) and vB_KpnM-VAC66 (query, 3%; identity, 85.05%), which are very similar (query, 95%; identity, 97.56%), as previously demonstrated in a study carried out by our research group (41) (Fig. 2).

### Host range assay.

**FIG 3:**
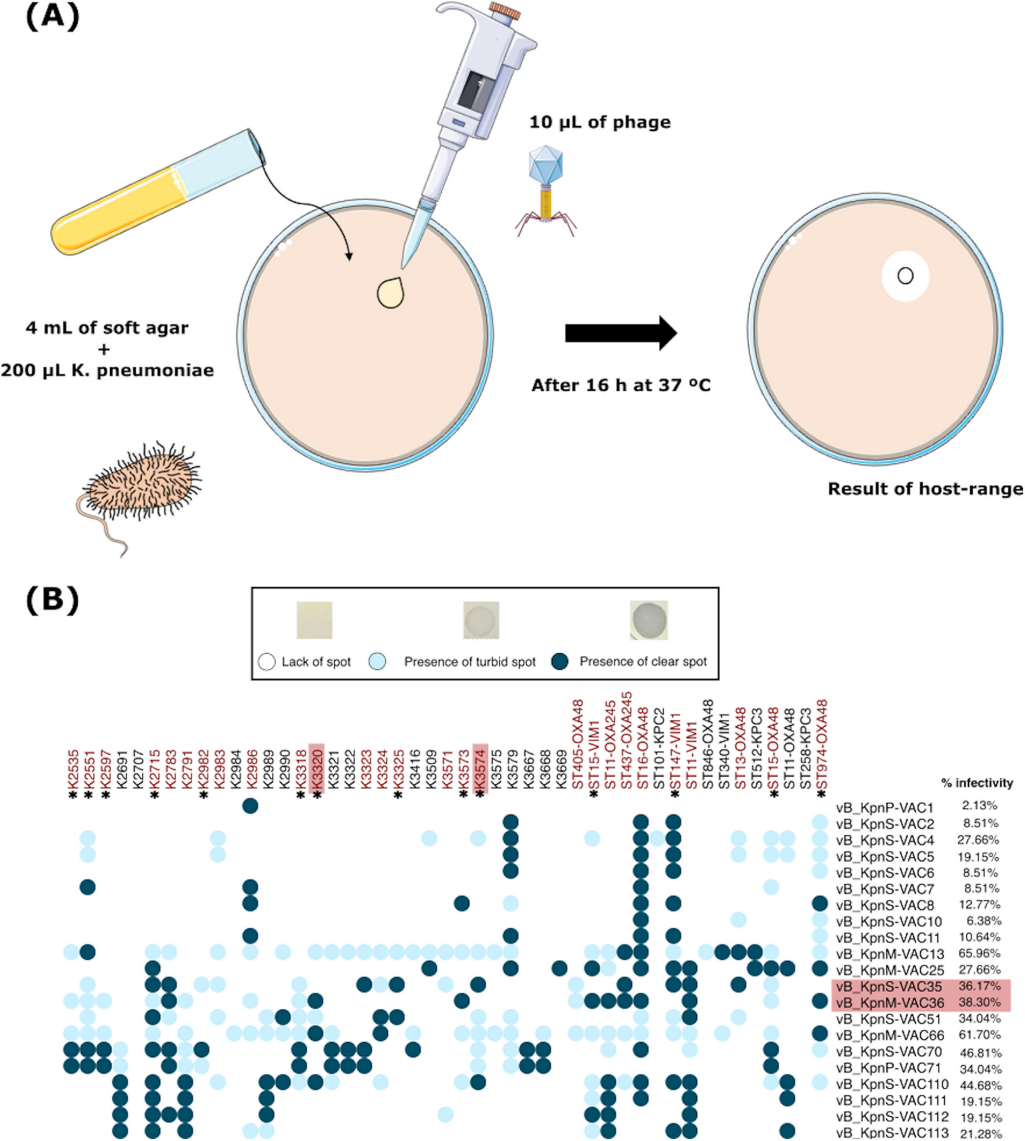
(A) Schematic representation of the host range technique. (B) Host range of the 21 phages included in the collection of 47 clinical strains of K. pneumoniae and the percentage of infectivity. The strains indicated in red are strains were infected by vB_KpnS-VAC35 and vB_KpnM-VAC36 phages, and an asterisk (*) represents the presence of the CRISPR-Cas system within these strains.

The phage infectivity assay was performed in the collection of 47 K. pneumoniae clinical strains (Table 3) by the spot test technique (Fig. 3A). The criteria used to determine the phage infectivity were the presence of clear spots (infection), the presence of turbid spots (low infection or resistance), and the lack of spots (no infection). The results showed a high variability of infectivity between the phages (Fig. 3B). Phage vB_KpnP-VAC1 had the lowest host range, infecting only the strain K2986, while phage vB_KpnM-VAC13 presented the highest range of activity, infecting 27 strains. Phages vB_KpnS-VAC35 and vB_KpnM-VAC36 also exhibited a high host range, infecting both 17 and 18 strains, respectively. Therefore, the following experiments focused on these two phages (vB_KpnS-VAC35 and vB_KpnM-VAC36).

**TABLE 3:** Characteristics of 47 clinical K. pneumoniae strains

Strain	MLST^*a*^	Carbapenemase(s)^*b*^	Capsular type(s)^*c*^	Origin	GenBank accession no.	Source or reference
K2535	ST15	SHV-28, SHV106	KL112	Blood	SAMEA3538911	This study
K2551	ST15	OXA-48, OXA-1, TEM-1B, CTX-M-15	KL112	Blood	SAMEA3538915	This study
K2597	ST15	OXA-48, OXA-1, TEM-1B, CTX-M-15	KL112	Blood	SAMEA3538926	This study
K2691	ST11	CTX-M-15	KL24	Blood	SAMEA3538940	40
K2707	ST11	KPC-2	KL13	Blood	SAMEA3538945	40
K2715	ST45	SHV-1	KL24	Blood	SAMEA3538948	This study
K2783	ST11	KPC-2	KL13	Blood	SAMEA3538957	This study
K2791	ST11	CTX-M-15	KL24	Blood	SAMEA3538958	This study
K2982	ST605	ND	KL58	Blood	SAMEA3649451	This study
K2983	ST2449	ND	KL5	Blood	SAMEA3649452	75
K2984	ST405	CTX-M-15, SHV-76, TEM-1B	KL151/K1151	Blood	SAMEA3649453	40
K2986	ST307	CTX-M-15	KL102	Blood	SAMEA3649454	40
K2989	ST661	OXA-1, SHV-27	KL24	Blood	SAMEA3649457	40
K2990	ST107	SHV-1	KL142	Blood	SAMEA3649458	This study
K3318	ST15	OXA-1, CTM-X-15, OXA-48, TEM-1B	KL112	Blood	SAMEA3649518	This study
**K3320** ^*d*^	**ST163**	**SHV-16**	**KL139**	**Blood**	**SAMEA3649520**	**This study**
K3321	ST466	SHV-33	KL22/K37	Blood	SAMEA3649521	This study
K3322	ST35	SHV-1	KL22/K37	Blood	SAMEA3649522	This study
K3323	ST3645	ND	KL126	Blood	SAMEA3649523	This study
K3324	ST542	SHV-1	KL8	Blood	SAMEA3649524	This study
K3325	ST42	ND	KL64	Blood	SAMEA3649525	40
K3416	ST483	SHV-27, VIM-1	KL110	Blood	SAMEA3649537	This study
K3509	ST35	SHV-1	KL22/K37	Blood	SAMEA3649551	This study
K3571	ST33	SHV-108	KL13	Blood	SAMEA3649557	This study
K3573	ST37	ND	KL15/K51/K52	Blood	SAMEA3649559	This study
**K3574**	**ST3647**	**ND**	**KL30**	**Blood**	**SAMEA3649560**	**This study**
K3575	ST14	SHV-1	KL2	Blood	SAMEA3649561	This study
K3579	ST16	CTX-M-15, OXA-1	KL51	Blood	SAMEA3649562	This study
K3667	ST326	ND	KL25	Blood	SAMEA3649564	This study
K3668	ST405	OXA-48, OXA-1, SHV-1	KL151/K1151	Blood	SAMEA3649629	This study
K3669	ST258	KPC-3	KL107/K81	Blood	SAMEA3649638	This study
ST405-OXA48	ST405	OXA-48	KL151/K1151	Wound	WRXJ00000000	2
ST15-VIM1	ST15	VIM-1	KL24	Blood	WRXI00000000	2
ST11-OXA245	ST11	OXA-245	KL24	Wound	WRXH00000000	2
ST437-OXA245	ST437	OXA-245	KL36	Rectal	WRXG00000000	2
ST16-OXA48	ST16	OXA-48	KL51	Urine	WRXF00000000	2
ST101-KPC2	ST101	KPC-2	KL17	Rectal	WRXE00000000	2
ST147-VIM1	ST147	VIM-1	KL64	Rectal	WRXD00000000	2
ST11-VIM1	ST11	VIM-1	KL24	Respiratory	WRXC00000000	2
ST846-OXA48	ST846	OXA-48	KL110	Sputum	WRXB00000000	2
ST340-VIM1	ST340	VIM-1	KL15	Rectal	WRXA00000000	2
ST13-OXA48	ST13	OXA-48	KL30	Rectal	WRWZ00000000	2
ST512-KP3	ST512	KPC-3	KL107/K81	Axillary	WRWY00000000	2
ST15-OXA48	ST15	OXA-48	KL112	Axillary	WRWX00000000	2
ST11-OXA48	ST11	OXA-48	KL24	Urine	WRWW00000000	2
ST258-KPC3	ST258	KPC-3	KL107/K81	Urine	WRWV00000000	2
ST979-OXA48	ST974	OXA-48	KL38	Urine	WRWT00000000	2

a MSLT, multilocus sequence type according to https://bigsdb.pasteur.fr/klebsiella/.

b Carbapenemase, as determined by https://bio.tools/resfinder.

c Capsular type, as determined by https://github.com/kelwyres/Kaptive-Web.

d Clinical strains selected for phage interaction studies are indicated in boldface.

### Bacterial genome analysis.

**FIG 4:**
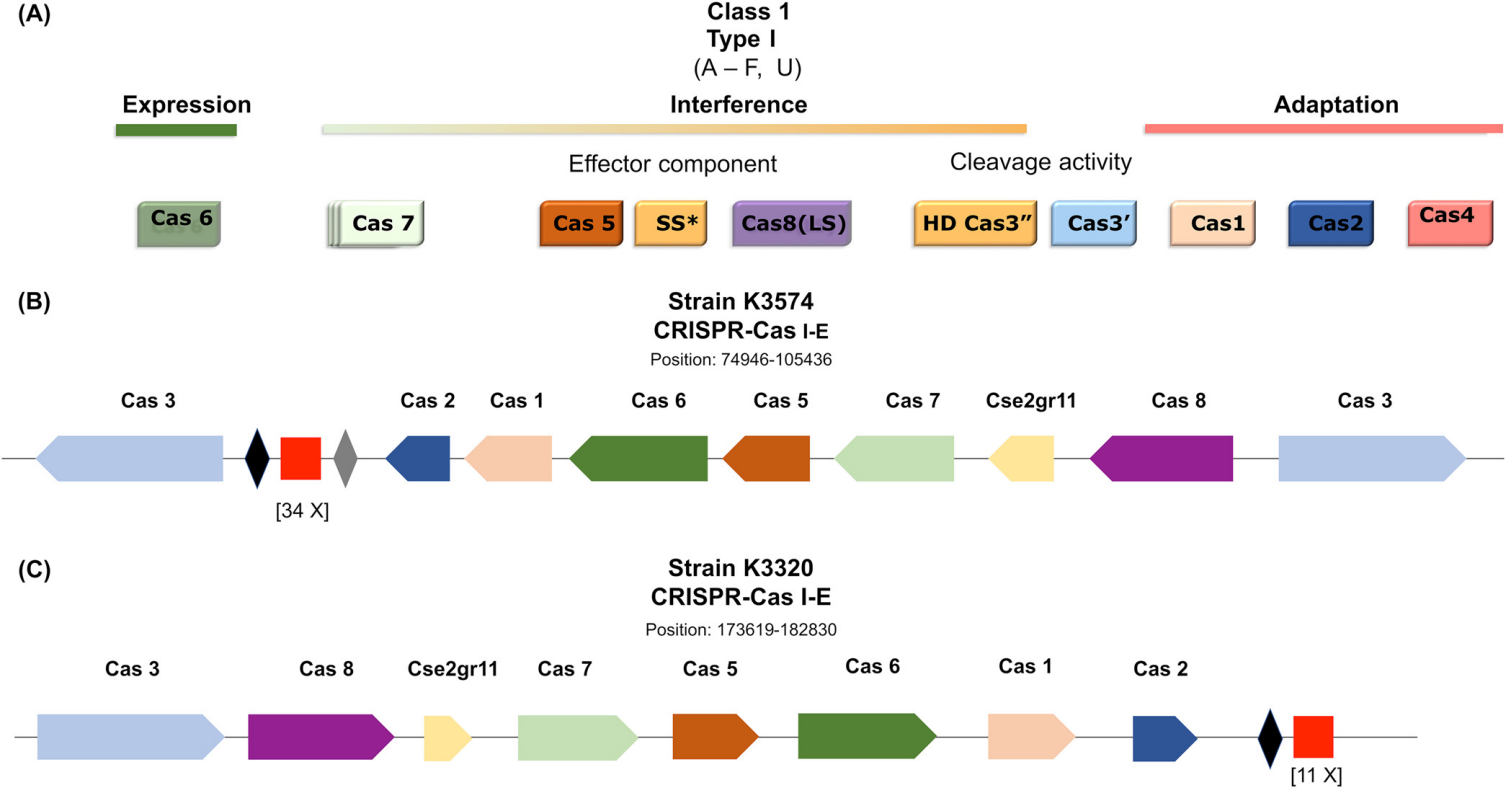
(A) Scheme of the modular organization of class I, type I CRISPR-Cas systems. (Diagram adapted from reference 74.) SS* indicates the putative small subunit (SS) that might be fused to the large subunit in several type I subtypes. (B and C) Graphic representation of the CRISPR-Cas system of strains K3574 and K3320, and adapted image of CRISPR Miner 2 (http://www.microbiome-bigdata.com/CRISPRminer, accessed in March 2022). The red square represents the repeats. Whereas the black and gray diamonds represent spacer sequences, the numbers below the repeats represent the numbers of spacers. The arrows represent the open reading frames, and the different colors represent the different Cas types.

The genomes of twenty-seven clinical isolates infected by the vB_KpnS-VAC35 and vB_KpnM-VAC36 phages, according to the host range assay, were analyzed for the presence of CRISPR-Cas systems. The result revealed the presence of CRISPR-Cas systems in 14 strains (Fig. 3B). However, we proceeded to a more detailed study of K. pneumoniae clinical strains K3574 (SAMEA3649560) and K3320 (SAMEA3649520), as they were the ones best infected by phages vB_KpnS-VAC35 and vB_KpnM-VAC36, respectively. The result of this study revealed that both strains had an intact type I-E CRISPR-Cas in their genome, with 35 spacers in the case of K3574 and with 11 spacers in the case of the strain K3320 (Fig. 4A and B). In turn, five plasmids located in five different contigs were found in the strain K3574, while strain K3320 had four plasmids located in three different contigs (Table 4). On the other hand, only strain K3574 exhibited an RM system, which was type II and functioned as a methyltransferase (Table 4). Finally, five prophages (two intact and three questionable) were detected in strain K3574, and seven prophages (three intact, two incomplete, and two questionable) were detected in strain K3320. However, only the data of the prophages considered intact are shown in Table 5.

**TABLE 4:** Plasmid and RM system of selected clinical K. pneumoniae strains K3574 and K3320

Database	Plasmid	Identity (%)	Contig	Position		Accession no.
				Start	Stop	
Plasmid						
K3574						
*Enterobacterales*	Col(pHAD28)	97.78	NODE_58_length_2240_cov_4.51213_ID_117	2148	2237	KU674895
*Enterobacterales*	Col440I	96.49	NODE_50_length_4243_cov_1686.76_ID_93	2628	2741	CP023920
*Enterobacterales*	IncFIA(HI1)	98.97	NODE_34_length_20733_cov_46.3618_ID_67	19450	19836	AF250878
*Enterobacterales*	IncFIB(K)	98.93	NODE_24_length_50018_cov_32.4049_ID_47	43034	43593	JN233704
*Enterobacterales*	IncFIB(pKPHS1)	96.43	NODE_1_length_470932_cov_21.7106_ID_1	156126	156685	CP003223
K3320						
*Enterobacterales*	Col(pHAD28)	100.0	NODE_100_length_2065_cov_2074.6_ID_199	1	100	KU674895
*Enterobacterales*	Col(pHAD28)	100.0	NODE_100_length_2065_cov_2074.6_ID_199	1886	2016	KU674895
*Enterobacterales*	IncFIB(K)	98.93	NODE_69_length_8285_cov_27.8857_ID_137	3503	4062	JN233704
*Enterobacterales*	IncR	99.2	NODE_76_length_6016_cov_46.5918_ID_151	648	898	DQ449578
RM (type: function)						
K3574						
Type II RM: methyltransferase	M.Kpn34618DCM: CCWGG^*a*^	99.79	Node_3_length_359415_cov_23.4099_ID_5	316287	317720	CPO10392 ^*b*^
K3320						
NA	NA	NA	NA	NA	NA	NA

a Gen: recognition sequences.

b Recognition sequences.

**TABLE 5:** Intact prophage found in the genomes of K3574 and K3320

Prophage	Region length (kb)	No. of total proteins	Contig	Position		%G+C
				Start	Stop	
K3574						
vB_Kpn-1.K3574	116.7	121	Node_1_length_470932_cov_21.7106_ID_1	132732	249436	50.1
vB_Kpn-2.K3574	34.7	46	Node_26_length_35850_cov_26.2256_ID_51	243	34952	51
						
K3320						
vB_Kpn-1.K3320	34.6	47	Node_6_length_198717_cov_16.8897_ID_11	32703	67316	55.3
vB_Kpn-2.K3320	55.9	82	Node_12_length_157528_Cov_17.5093_ID_23	338	56297	50.2
vB_Kpn-3.K3320	27.1	39	Node_45_length_28501_Cov_18.8915_ID_89	842	27971	51.2

### Characterization of phages vB_KpnS-VAC35 and vB_KpnM-VAC36.

**FIG 5:**
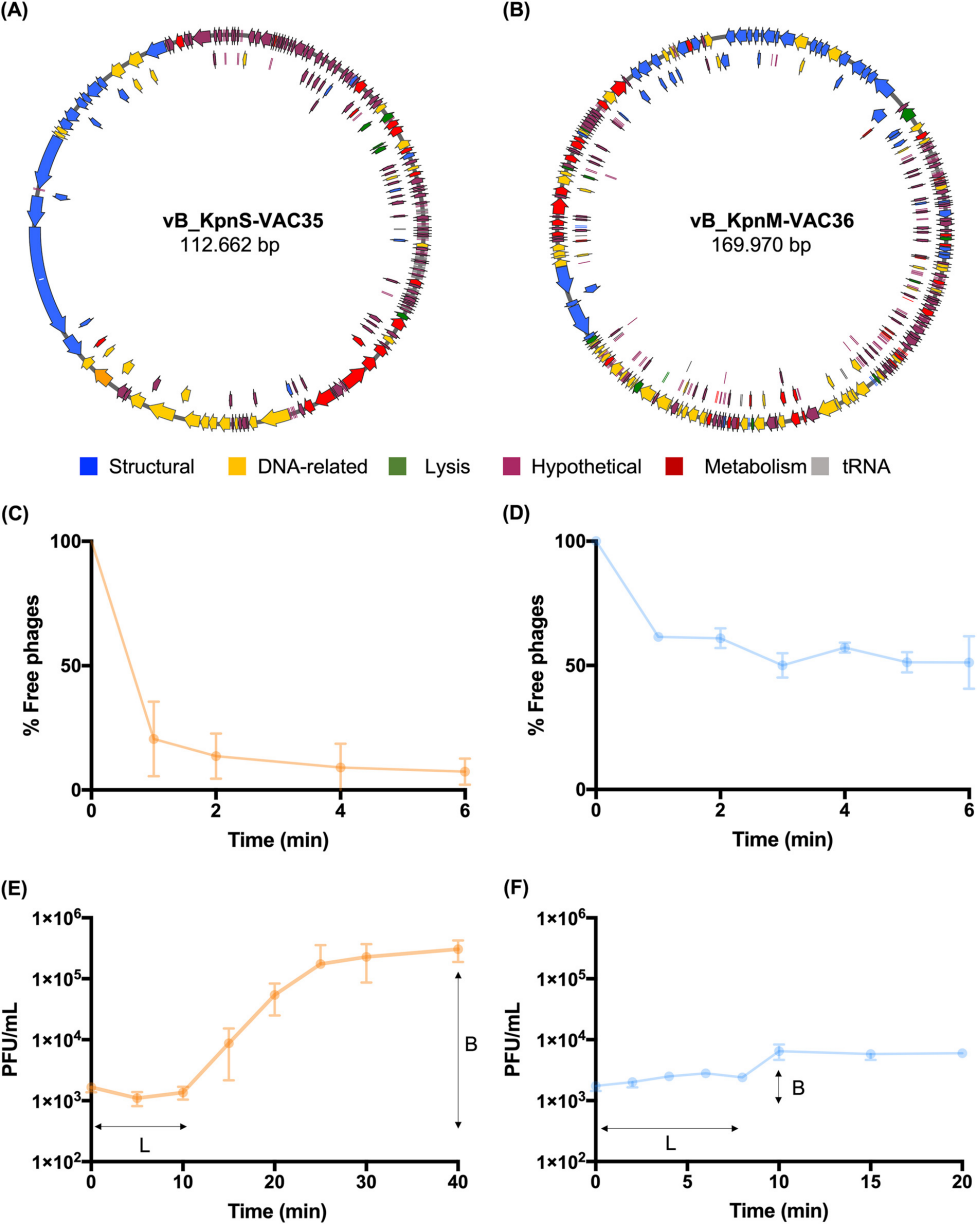
(A and B) Graphic representation of the genome of the phage vB_KpnS-VAC35 and vB_KpnM-VAC36, constructed with the Snapgene tool, v6.0.5. (C and D) Adsorption curve of phages vB_KpnS-VAC35 and vB_KpnM-VAC36, with adsorption times of 5 and 2 min, respectively. The error bars represent the standard deviations of three experimental replicates. (E and F) One-step growth curve of phages vB_KpnS-VAC35 and vB_KpnM-VAC36, with latent times (labeled “L”) of 10 and 8 min and a burst size (labeled “B”) of 45.52 PFU/mL and 2.71, respectively. The error bars represent the standard deviations of three experimental replicates.

**(i) Phage adsorption.** Adsorption of phages vB_KpnS-VAC35 and vB_KpnM-VAC36 (Fig. 5A and B) to the bacterial surface receptor was studied with the previously selected strains K3574 and K3320, at a multiplicity of infection (MOI) of 0.01. Phage vB_KpnS-VAC35 showed a high percentage of adsorption, with 91.28% of phage adsorbed in the strain K3574 after 5 min, while phage vB_KpnM-VAC36 showed slight adsorption in the strain K3320, with 39.02% of phage adsorbed after 2 min (Fig. 5C and D).

**(ii) One-step growth curve assay.** The latent period, determined by the one-step growth curve indicating the time taken for a phage particle to reproduce inside an infected host cell, and the burst size, defined as the number of viral particles released in each infection cycle per cell, were 10 min and 45.52 PFU/mL, respectively, for phage vB_KpnS-VAC35 in strain K3574 and 8 min and 2.71 PFU/mL for the phage vB_KpnM-VAC36 in strain K3320 at an MOI of 0.01 (Fig. 5E and F).

**FIG 6:**
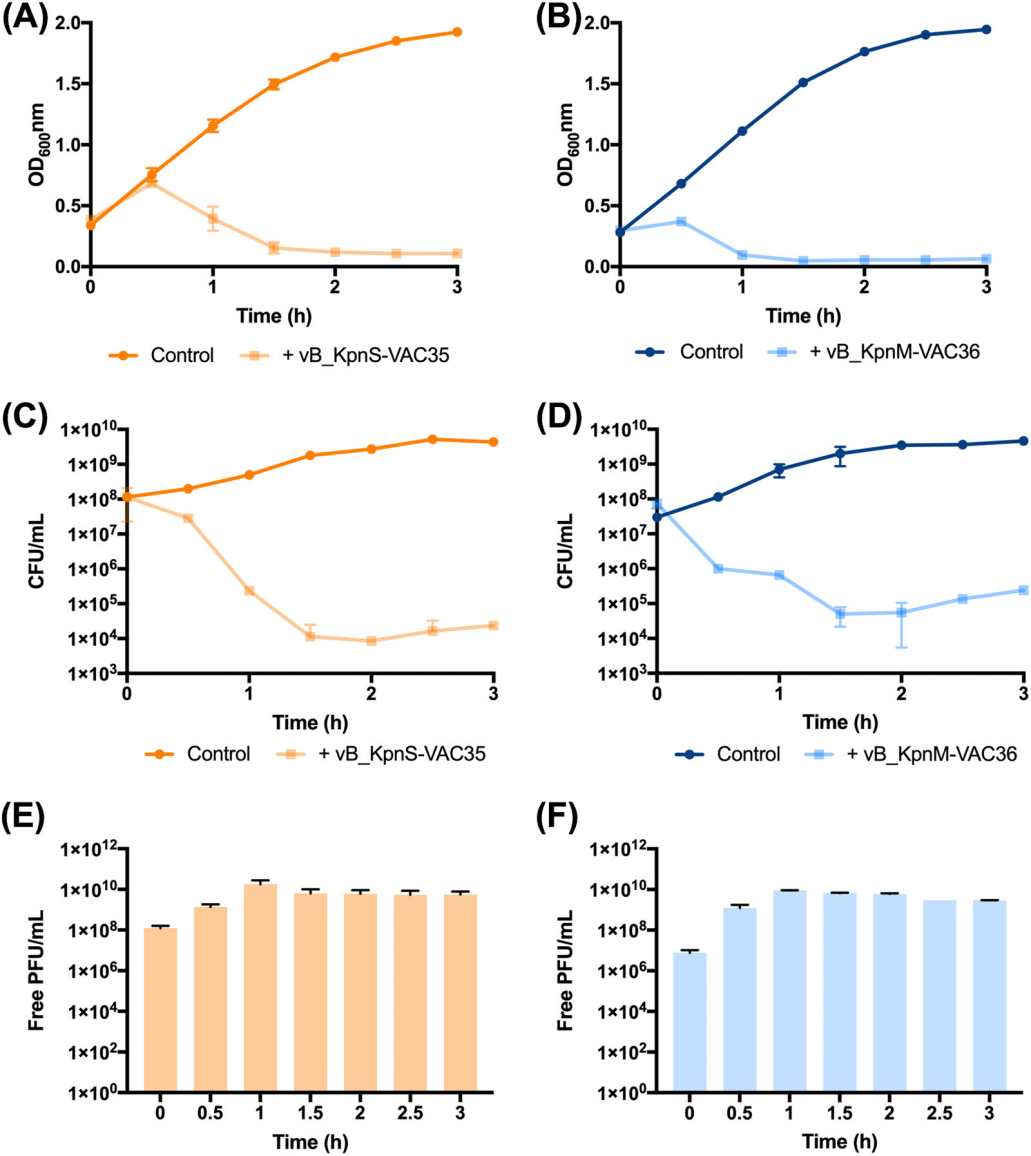
(A and B) Infection curve of the strains K3574 (orange) and K3320 (blue), respectively, with phages vB_KpnS-VAC35 (light orange) and vB_KpnM-VAC36 (light blue) at an MOI of 1. (C and D) Measurement of viability by CFU/mL counts of strains K3574 and K3320 infected with, respectively, phage vB_KpnS-VAC35 and vB_KpnM-VAC36, at an MOI of 1 over time. (E and F) Measurement of PFU/mL counts of the phages vB_KpnS-VAC35 and vB_KpnM-VAC36 at an MOI 1 over time.

**(iii) Phage kill curve.** The infectivity assay in liquid medium to determine the infection curve for phages vB_KpnS-VAC35 and vB_KpnM-VAC36 at an MOI of 1 in the selected clinical strains: K3574 and K3320, showed that both phages yielded successful infection, with optical density at 600 nm (OD_600_) values of 0.155 ± 0.05 and 0.05 ± 0.01, respectively, reached after 1 h 30 min of infection (Fig. 6A and B). We monitored the CFU/mL and observed that, for phage vB_KpnS-VAC35 in clinical strain K3574, the count reached 1.15 × 10^4^ ± 1.34 × 10^4^ CFU/mL. However, we observed a slight increase in the number of CFU/mL after 3 h of phage infection; it reached 2.35 × 10^4^ ± 2.12 × 10^4^ CFU/mL. The same phenomenon appears in the case of the phage vB_KpnM-VAC36, where we observed a slight decrease in CFU/mL counts, which reached a value of 5.0 × 10^4^ ± 2.83 × 10^4^ after 1 h 30 min of phage infection and an increase of count after 2 h 30 min of phage infection 1.35 × 10^5^ ± 3.54 × 10^4^ (Fig. 6C and D). Thus, although the appearance of resistant bacteria was not observed in the OD test and the density remained unchanged for both phages, the bacteria did appear in the CFU/mL count test. Finally, we monitored the PFU/mL, and in both cases observed an increase in the number of PFU/mL after 30 min of phage infection, with values of 1.25 × 10^9^ ± 4.95 × 10^8^ PFU/mL for the phage vB_KpnS-VAC35 and 1.35 × 10^9^ ± 4.95 × 10^8^ PFU/mL for the phage vB_KpnM-VAC36 (Fig. 5E and F). Consequently, we can conclude that the number of CFU/mL is inversely proportional to the number of PFU/mL. These data confirm that the reduction in CFU is due to multiplication of the phages.

### NanoUHPLC-Tims-QTOF proteomic analysis: interaction between phages (vB_KpnS-VAC35 and vB_KpnM-VAC36) and clinical strains (K3574 and K3320).

**FIG 7:**
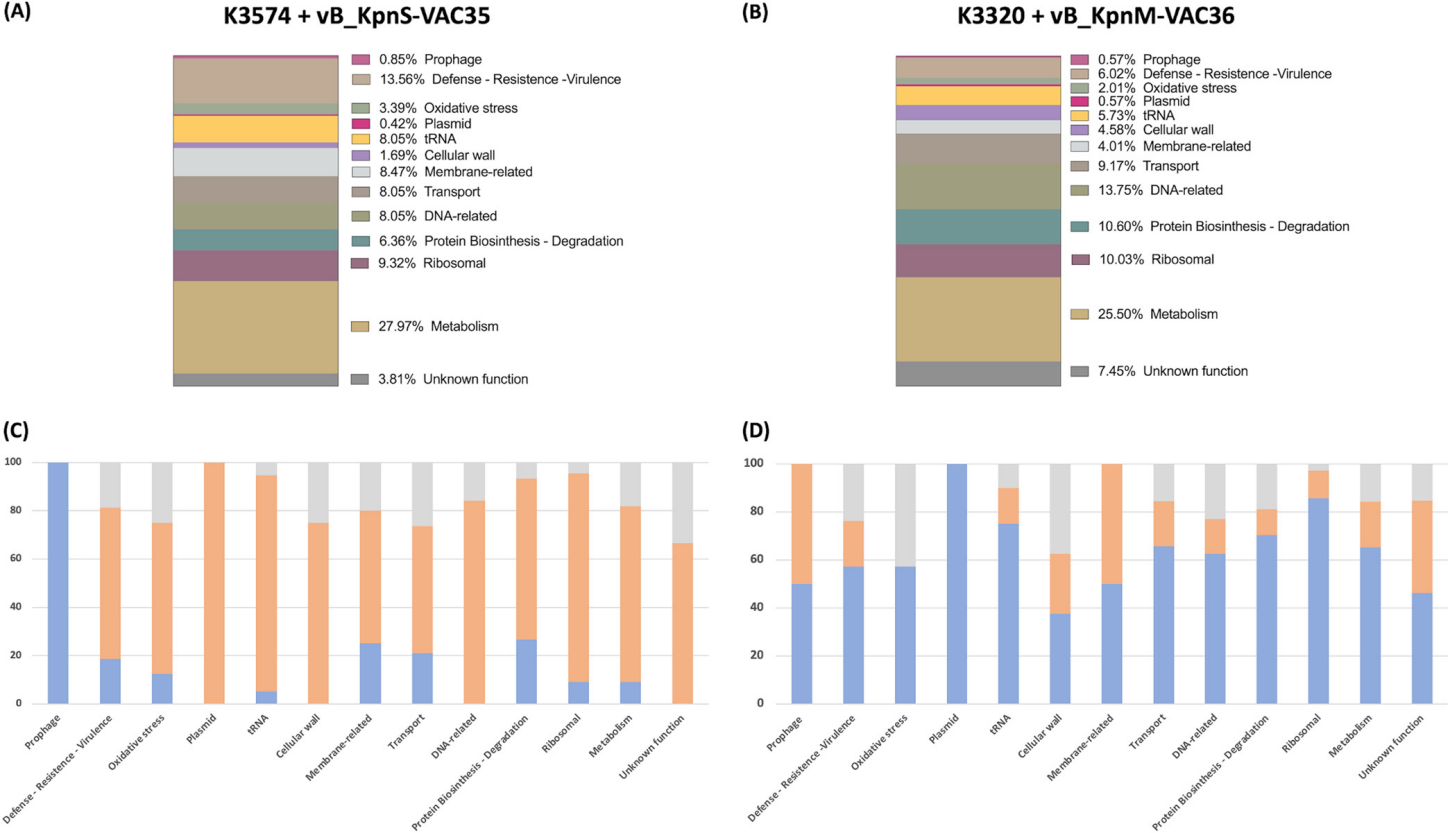
(A and B) Graphical representation of the proteomics results, showing the abundance of each group of proteins found in the culture with the bacterial strain K3574 infected with phage vB_KpnS-VAC35 and in the culture with the bacterial strain K3320 infected with phage vB_KpnM-VAC36. (C and D) Abundance of proteins with a higher (blue), lower (orange), or undetected (gray) value areas compared to the uninfected control in strains K3574 and K3320 infected with phages vB_KpnS-VAC35 and vB_KpnM-VAC36, respectively.

The proteomic study conducted by NanoUHPLC-Tims-QTOF (20) analysis revealed a large variety of proteins (listed in Fig. 7A and B, with the respective proportions in each strain): prophage-related proteins, defense, resistance and virulence proteins, oxidative stress proteins, plasmid-related proteins, tRNA, cell wall-related proteins, and membrane proteins, as well as some transport proteins and proteins related to DNA, biosynthesis or degradation of proteins, ribosomes, metabolism, and some of unknown function showing differences in expression compared to the uninfected control (Fig. 7C and D). In both cases, some prophage-related proteins, as well as a large amount of tRNA, were found. Regarding the defense proteins, we found proteins related to porins, multidrug efflux RND transporter, RM system type I methyltransferase, two-component response regulator system, TA system type II RelE/ParE family, DNA starvation protein, fimbriae, and finally pili. The details of all detected proteins showing differences from the control are summarized in Table S2 in the supplemental material. The presence of some Acr candidates in the phage-infected strains was detected by NanoUHPLC-Tims-QTOF analysis: seven in K3574 infected by vB_KpnS-VAC35 and one in K3320 infected by the vB_KpnM-VAC36 phage (Table 6).

**TABLE 6:** Acr candidate protein in phages vB_KpnS-VAC35 and vB_KpnM-VAC36 detected in the proteomic study (NanoUHPLC-Tims-QTOF)^*a*^

Description	Accession no.	–10LogP	Position(s)
K3574+vB_KpnS-VAC35			
Hypothetical protein KPN4_89 (Acr candidate) [Klebsiella phage KPN4]	QEG11275.1	146.67	24497–24667
4-Hydroxy-3-polyprenylbenzoate decarboxylase (Acr candidate) [Klebsiella phage vB_KpnS-VAC35]	UEP19035.1	107.65	22800–233162
Hypothetical protein JIPhKp127_0059 (Acr candidate) [Klebsiella phage JIPh_Kp127]	QFR57489.1	104.09	23369–23911
6-Phosphofructokinase (Acr candidate) [Klebsiella phage vB_KpnS-VAC35]	UEP19028.1	102.81	20466–20858
Hypothetical protein (Acr candidate) [Klebsiella phage vB_KpnS-VAC35]	UEP19032.1	86.070	22064–22273
Hypothetical protein (Acr candidate) [Klebsiella phage vB_KpnS-VAC35]	UEP19030.1	66.120	21405–21677
Hypothetical protein (Acr candidate) [Klebsiella phage vB_KpnS-VAC35]	UEP19036.1	61.440	23155–23382
			
K3320 + vB_KpnM-VAC36			
Hypothetical protein (Acr candidate) [Klebsiella phage vB_KpnM-VAC36]	UEP19294.1	78.95	66295–66492

a The protein header information is presented as listed in the NCBI database. The accession number of the protein as seen in the NCBI database is indicated. “–10LogP” indicates the protein confidence score, and “position(s)” indicates localization in the phage genome.

## DISCUSSION

Lytic phage therapy is currently considered one of the best alternatives for treating infections caused by MDR bacterial pathogens (3, 4). Phages are known to exhibit some advantages over the use of antibiotics, including the continued warfare between phage and bacteria during the coevolution of both organisms (12). Consequently, phages have developed defense mechanisms to evade the resistance mechanisms of bacteria (26–38), while at the same time bacteria have developed defense mechanisms to prevent phage infection (43). In this context, the aims of the present study were to analyze 21 new lytic phages in search of defense mechanisms and also to identify the defense mechanisms of two clinical strains K3574 and K3320 when infected by phages, since better knowledge of the latter will lead to improvements in the use of phages to treat infections caused by MDR bacteria.

Regarding the results of the whole-genome sequencing (WGS) and annotation, we observed that all phages belonged to the order *Caudovirales*. Several studies have shown that dsDNA-tailed phages are the most abundant entity on earth (44, 45). Most of these phages (61.90%) are members of the *Drexerviridae* family, 14.29% are members of the *Autographiviridae* family, 14.29% are members of the *Myoviridae* family, and 9.52% are members of the *Demerecviridae* family. Genome annotation has previously shown that all phages are lytic and lacking lysogenic genes such as integrase, recombinase, and excisionase (20). This point is of vital importance for use of these phages in phage therapy (46, 47). Most phages were found to have a typical organization of the genome in functional modules, as previously described (2, 48, 49). In contrast, members of the *Myoviridae* family, which are included in the “larger phages” (>100 bp), did not present specific lysis blocks, and structural and morphogenesis-related proteins were repeated in several blocks throughout the genome (41, 50). The genomes of all phages had endolysins and holins, proteins that are responsible for degradation of the bacterial cell wall during the infection by the host to facilitate the exit of the phage progeny (51).

Genomic annotation revealed the presence of numerous bacterial defense mechanisms: RM system evasion, TA system, DNA degradation evasion, blocking RM of host bacteria, genes that confer resistance to Abi system of host bacteria, a possible orphan CRISPR-Cas system, and almost all the phages possessed a possible anti-CRISPR system. These mechanisms have all already been described (14). The anti-CRISPR, which is composed by operons of Acr and Aca proteins, was first discovered in 2013 in phages and prophages of Pseudomonas aeruginosa (52). Acr-Aca operons are defined as genomic loci fulfilling the following criteria: (i) all genes should be in the same strand, (ii) all intergenic distances should be <150 bp, (iii) all genes encode proteins shorter than 200 amino acids in length, and, finally, (iv) at least one gene should be homologous to Acr or Aca proteins (53). The main problem of the search of new anti-CRIPSR is that Acr proteins are very poorly conserved, and the best way to discover new anti-CRISPR is therefore to use a “guilt-by-association” approach, which searches for Aca in the genome of phages. Although the function of Acas is not yet understood, these gene often encode a protein containing a helix-turn-helix motif, suggesting that they fulfill a regulatory function (54).

The study of phage infective capacity revealed a large disparity in the infectivity, as previously demonstrated (55, 56): phages vB_KpnM-VAC13 and vB_KpnM-VAC66 displayed the highest infectivity capacity (41), whereas phage vB_KpnP-VAC1 displayed the lowest infective capacity (20). The wide host range could be an advantage since it allows infection of a larger number of hosts (57), and this trait could be useful for successful phage therapy. In addition, the “larger phages” vB_KpnS-VAC35 (112.662 bp) and vB_KpnM-VAC36 (169.970 bp) showed a high infectivity for 17 and 18 clinical strains, respectively. Moreover, a possible anti-CRISPR system was detected in their genome. Thus, the presence of the CRISPR-Cas system in the bacterial strains that were successfully infected by these phages was examined to study the possible interaction of both defense mechanisms. The result of this search showed the presence of class I type I-E intact CRISPR-Cas system in the genome of the strain K3574 and K3320. Both phages were examined with their respective host strains. The adsorption curve revealed that phage vB_KpnS-VAC35 displays a higher percentage of adsorption, a higher burst size, and a longer latent period than phage vB_KpnM-VAC36. Moreover, analysis of the infectivity capacity by killing assay measuring the OD_600_, CFU/mL, and PFU/mL revealed that phage vB_KpnS-VAC35 was more effective than phage vB_KpnM-VAC36.

Finally, proteomic studies were conducted with bacterial strains K3574 and K3320 with or without phage infection (vB_KpnS-VAC35 and vB_KpnM-VAC36) to determine any differences at the level of protein expression after phage infection. The pattern of protein expression was found to vary depending on the strain considered. This may be due to the different infection status of the bacterial cell at the time of sample processing or due to the inherent proprieties of the bacteria. Therefore, the results revealed the expression of FtsH protease modulator located in prophage of strains K3574 and K3320, which controls the lytic pathway (58), as well as the expression of the cupin protein located in plasmid in the strain K3574, a phosphomannose isomerase involved in lipopolysaccharide (LPS) synthesis, which is an important determinant of pathogenicity and phage susceptibility (59). In addition, proteins related to bacterial defense, resistance, and virulence and also to oxidative stress mechanisms (60) have been observed. The difference in expression compared to the control without phage infection of porins, efflux pumps, LPS, and pilus elements, previously described in the literature as phage receptors (61, 62), was also observed. Moreover, proteins involved in the *quorum* network were observed, e.g., the LuxS that synthesizes AI-2 molecules, or the presence of the CsrA regulator in the strain K3320 infected by phage vB_KpnM-VAC36 (63, 64). Indeed, previous studies have associated the quorum network with phage infection (65–67). In addition, a type II RelE/ParE TA system was expressed in strain K3320. This is a very interesting finding, since phage vB_KpnM-VAC36 did not successfully infect strain K3320. The fundamental role played by TA systems in the inhibition of phage infection has recently been demonstrated (20, 68–70). Interestingly, an inhibitor of the TA system (protein ID QZE51102.1) was found in the genome of the phage vB_KpnP-VAC1. This type of gene, previously only described in one E. coli phage (35), may play a role in phage defense against bacteria.

The methyltransferases, other important proteins that play a key role in phage infection (71), were found in both K3574 and K3320 strains infected by phages. In addition, several Acr candidate proteins were expressed in the infected strains. This is a very interesting finding, because the anti-CRISPR could inhibit the host’s CRISPR-Cas system and thus promote infection (54). Therefore, the difference in expression compared to the control of all mechanisms could be due to the phage-host interaction, with the bacteria trying to use all their defense mechanisms in response to the infection.

### Conclusion.

Phage-host interactions have been examined ever since the discovery of phages a century ago. The present study revealed numerous defense mechanisms both against bacteria by phage (RM system evasion, TA system, DNA degradation evasion, RM block of host, resistance to Abi, anti-CRISPR and CRISPR-Cas system) and against phage infection by bacteria (prophage, plasmid, defense/virulence/resistance, and oxidative stress proteins). However, phage-host bacterial interactions remain poorly understood, and further study is required in order to improve the efficacy of phage therapy.

## MATERIALS AND METHODS

### Bacterial strains.

A collection of 47 clinical isolates of K. pneumoniae obtained from the Virgen Macarena University Hospital (Seville, Spain) and the National Centers for Microbiology (Carlos III Health Institute, Spain) was used in this study (Table 3). The sequence type (ST) and the capsular type (K) were determined using the methods available on the Pasteur Institute website (https://bigsdb.pasteur.fr/klebsiella/, accessed between 2018 and the present) and the Kaptive website (https://github.com/kelwyres/Kaptive-Web, accessed in April 2020), respectively. All strains were grown in Luria-Bertani medium (0.5% NaCl, 0.5% yeast extract, 1% tryptone).

### Isolation, purification, and propagation of lytic phages and TEM.

Ten new lytic phages isolated from sewage water samples and twelve lytic phages previously isolated by our research group (20, 40, 41) were used in this study. Isolation, purification, and propagation of the new phages was performed according to the procedures used in the previously cited articles, using strains of K. pneumoniae as a natural host (Table 1). Next, the 10 new lytic phage solutions were negatively stained with 1% aqueous uranyl acetate before being analyzed by TEM in a JEOL JEM-1011 electron microscope.

### Phage DNA extraction and WGS.

The phage DNA of the 10 new lytic phages was isolated with the phenol-chloroform method according to a previously published phage-hunting protocol (https://phagesdb.org/media/workflow/protocols/pdfs/PCI_SDS_DNA_Extraction_2.2013.pdf, accessed on 1 February 2021), and WGS was performed as described by Bleriot et al. (20).

### Phage genome annotation.

**(i) Defense mechanisms.** All assemblies were initially annotated by sequence homology using Patric 3.6.9 (http://patricbrc.org, accessed on 22 February 2021) and were then manually refined using BLASTX (http://blast.ncbi.nlm.nih.gov, accessed between August and October 2021) and Hhmer (http://hmmer.org, accessed between August and October 2021), as well as the Hhpred tool (https://toolkit.tuebingen.mpg.de/tools/hhpred, accessed between August and October 2021), which predict functions through protein structure. In addition, to search for phage defense mechanisms against bacteria, the CRISPR Miner 2 (http://www.microbiome-bigdata.com/CRISPRminer/, accessed in March 2022) and PADLOC (https://padloc.otago.ac.nz/padloc/, accessed in March 2022) tools were used to search for possible CRISPR-Cas systems, as well as the AcrDB tool (https://bcb.unl.edu/AcrFinder/, accessed on October 2021) to search for possible anti-CRIPSR-cas systems with the defect parameter of the website (Aca E value, 0.01; Aca identity, 30%; Aca coverage, 0.8; maximum intergenic distance between genes [bp], 150; operon up/downstream range for MGE-Prophage search [no. of genes], 10). Finally, the family and genus of the different phages were determined by sequence homology with the phage sequences available in the NCBI database. Complete genome sequences were included in the GenBank BioProject PRJNA739095.

**(ii) Phage phylogenetic analysis and genome comparison.** Phylogenetic analysis of the 21 phages was performed using the nucleotide sequence of the large terminase subunit of each phage. Alignment was first performed with MAFF server (https://mafft.cbrc.jp/alignment/server/index.html, accessed on 3 January 2022), and a phylogenetic tree was then constructed using RAxMLHPC-PTHREADS-AVX2 v8.2.12 (42) under the GTRGAMMA model and 100 bootstrap replicates. A graphical representation of the comparison of all phage genomes was then constructed with the VipTree website (https://www.genome.jp/viptree/, accessed in June 2022) according to the previously established phylogenetic relationship.

### Host range assay.

The phage host spectrum was tested by the spot test technique (72), in a collection of 47 clinical strains of K. pneumoniae. A negative control consisting of SM buffer (0.1 M NaCl, 10 mM MgSO_4_, 20 mM Tris-HCl; pH 7.5) was included in each plate. All determinations were made in triplicate. The criteria used to determine the phage infectivity were the presence of clear spots (infection), the presence of turbid spots (low infection or resistance), and the lack of spots (no infection).

### Study of bacterial genome.

The genome of 27 clinical isolates infected by the vB_KpnS-VAC35 and vB_KpnM-VAC36 phages, according to the host range assay, were analyzed for the presence of CRISPR-Cas systems by using the CRISPR Miner 2 (http://www.microbiome-bigdata.com/CRISPRminer/, accessed in January 2023). However, we proceeded to a more detailed study of K. pneumoniae clinical strains K3574 (SAMEA3649560) and K3320 (SAMEA3649520), since it was the one showing the best phage infectivity; for this, the genomic annotation of the Rastserver was also studied to check and validate the results obtained with other tools (https://rast.nmpdr.org, accessed in August 2022). Plasmids were then searched for using PlasmidFinder v2.0.1 (20 July 2001) (https://cge.food.dtu.dk/services/PlasmidFinder/, accessed in July 2022), the RM system using the Restriction-ModificationFinder v1.1 (accessed in June 2015) (https://cge.food.dtu.dk/services/Restriction-ModificationFinder/history.php, accessed in July 2022), and prophages using the Phaster tools (http://phaster.ca, accessed in July 2022).

### Characterization of phages vB_KpnS-VAC35 and vB_KpnM-VAC36.

**(i) Phage adsorption.** Adsorption of phages vB_KpnS-VAC35 and vB_KpnM-VAC36 to the bacterial surface receptors of clinical strains K3574 and K3320, respectively, was determined from the adsorption curve (73) at an MOI of 0.01. The number of phages mixed with bacterial host cells at time zero was considered 100% free of phages. The adsorption curve analysis was performed in triplicate.

**(ii) One-step growth curve assay.** A one-step growth curve of phages vB_KpnS-VAC35 and vB_KpnM-VAC36 at an MOI of 0.01, was constructed in clinical strains K3574 and K3320, respectively, to determine the latent period (L) and the burst size (B), according to the procedure of Bleriot et al. (20). One-step growth curve analysis was performed in triplicate.

**(iii) Phage kill curve assay in liquid medium.** Killing curves were constructed from the selected isolates K3574 and K3320, in accordance with the presence of intact CRISPR-Cas system in their genome. Phages vB_KpnS-VAC35 and vB_KpnM-VAC36 were used to monitor the infection of the strain by optical density measured at a wavelength of 600 nm (OD_600_) and counts of CFU/mL and PFU/mL. For this purpose, when the strains reached an early logarithmic phase (OD_600_ = 0.3 to 0.4), cultures were infected with phages at an MOI of 1. The OD_600_, the CFU/mL, and the PFU/mL were determined every 30 min for 3 h. In all cases, the control was the strain without phage infection. All analyses were performed in triplicate.

### NanoUHPLC-Tims-QTOF proteomic analysis: interaction between phages (vB_KpnS-VAC35 and vB_KpnM-VAC36) and clinical strains (K3574 and K3320).

NanoUHPLC-Tims-QTOF analysis was performed for quantitative study of the protein profile of strain K3574 and K3320 with or without infection with phages vB_KpnS-VAC35 and vB_KpnM-VAC36. When the cultures reached an early logarithmic phase of growth (OD_600_ = 0.3 to 0.4), they were infected with phages at an MOI of 1. After 1 h, the cultures of the strains were harvested by centrifugation at 4,302 × *g* for 20 min at 4°C. The pellets were then stored at −80°C to facilitate cell disruption. The next day, the pellet was resuspended in phosphate-buffered saline and sonicated. Finally, the sonicated pellets were centrifuged at 4,302 × *g* for 20 min at 4°C, and the flowthrough was analyzed using NanoUHPLC-Tims-QTOF. The equipment, as well as the procedure used, are as described by Bleriot et al. (20).

### Data availability.

Phage genome sequencing revealed all phages under study (available from GenBank BioProject PRJNA739095).
