# Supplementary material for: Proteomic Study of the Interactions between Phages and the Bacterial Host Klebsiella pneumoniae
Source: Microbiol Spectr. 2023 Mar 6;11(2):e03974-22. doi: 10.1128/spectrum.03974-22 (PMC10100988; doi:10.1128/spectrum.03974-22)
Supplement: Supplemental file 3 — Legends of Tables S1 and S2. Download spectrum.03974-22-s0001.pdf, PDF file, 0.05 MB [file spectrum.03974-22-s0001.pdf]

SUPPLEMENTARY FILES

Supplementary Table 1. Possible Aca and Acr proteins of the anti-CRISPR system detected in the phages with the AcrDB bioinformatics tool (https://bcb.unl.edu/AcrFinder/, accessed in October 2021).

Supplementary Table 2. Table of the proteins found in the proteomic study by NanoUHPLC-Tims-QTOF in the strains infected with phages vB_KpnS-VAC35 and vB_KpnM-VAC36 and in the control without phage infection. *Description* the protein header information as seen in the NCBI database, *-10LogP* the protein confidence score. *Area* the area under the curve of the peptide feature found at the same *m/z* and retention time as the MS/MS scan. This can be used as an indicator of the abundance and *Avg. Mass* is the protein mass calculated using the average mass. The area values of the phage-infected strain that are coloured red mean that they are lower than the control values. The area values for the phage-infected strain in blue mean that they are higher than the control values. The area values for the phage-infected strain in bold mean that the proteins are not detected in the control.
